# Disappearance of an ecosystem engineer, the white-lipped peccary (*Tayassu pecari*)*,* leads to density compensation and ecological release

**DOI:** 10.1007/s00442-022-05233-5

**Published:** 2022-08-13

**Authors:** Andrew Whitworth, Christopher Beirne, Arianna Basto, Eleanor Flatt, Mathias Tobler, George Powell, John Terborgh, Adrian Forsyth

**Affiliations:** 1 Osa Conservation, Washington, DC USA; 2 Institute of Biodiversity, Animal Health and Comparative Medicine, College of Medical, Veterinary and Life Sciences, University of Glasgow, Glasgow, Scotland UK; 3 Department of Biology, Center for Energy, Environment, and Sustainability, Wake Forest University, Winston-Salem, NC USA; 4 Department of Forest Resources Management, University of British Columbia, Vancouver, Canada; 5 Conservación Amazónica, Lima, Perú; 6 Human Dimensions of Natural Resources, Colorado State University, Fort Collins, CO USA; 7 San Diego Zoo Wildlife Alliance, San Diego, CA USA; 8 Wildlife Protection Solutions, Denver, CO USA; 9 Nicholas School of the Environment, Duke University, Durham, NC USA; 10 Andes Amazon Fund, Washington, DC USA

**Keywords:** Community ecology, Amazon rainforest, Trophic interactions, Jaguar, Camera traps

## Abstract

Given the rate of biodiversity loss, there is an urgent need to understand community-level responses to extirpation events, with two prevailing hypotheses. On one hand, the loss of an apex predator leads to an increase in primary prey species, triggering a trophic cascade of other changes within the community, while density compensation and ecological release can occur because of reduced competition for resources and absence of direct aggression. White-lipped peccary (*Tayassu pecari*—WLP), a species that typically co-occurs with collared peccary (*Pecari tajacu*), undergo major population crashes—often taking 20 to 30-years for populations to recover. Using a temporally replicated camera trapping dataset, in both a pre- and post- WLP crash, we explore how WLP disappearance alters the structure of a Neotropical vertebrate community with findings indicative of density compensation. White-lipped peccary were the most frequently detected terrestrial mammal in the 2006–2007 pre-population crash period but were undetected during the 2019 post-crash survey. *Panthera onca* (jaguar) camera trap encounter rates declined by 63% following the WLP crash, while collared peccary, puma (*Puma concolor*), red-brocket deer (*Mazama americana*) and short-eared dog (*Atelocynus microtis*) all displayed greater encounter rates (490%, 150%, 280%, and 500% respectively), and increased in rank-abundance. Absence of WLP was correlated with ecological release changes in habitat-use for six species, with the greatest increase in use in the preferred floodplain habitat of the WLP. Surprisingly, community-weighted mean trait distributions (body size, feeding guild and nocturnality) did not change, suggesting functional redundancy in diverse tropical mammal assemblages.

**Supplementary Information:**

The online version contains supplementary material available at 10.1007/s00442-022-05233-5.

## Introduction

Characterising how communities respond to the loss of species is a fundamental challenge in ecology with the potential to lend insights into the mechanisms governing species diversity (Terborgh 2015). This is particularly important within the Anthropocene, as range contractions and local extirpations are occurring at unprecedented rates (Turvey and Crees 2019). There are two prevailing hypotheses about the consequences of extirpation on faunal communities. On one hand, the loss of a single apex predator leads to an increase in primary prey species, which often triggers a trophic cascade of other changes and/or extirpations within the community (Dunne and Williams 2009; Brodie et al. 2014). Conversely, density compensation and ecological release—in the sense of Hermann et al. (2021)—can occur as a result of reduced competition for resources and absence of direct aggression (Ritchie and Johnson 2009). Whereas trophic cascades have the potential to markedly alter a community’s functional composition, with consequences for ecological processes, functions, and services (Cadotte et al. 2011), density compensation and ecological release may buffer ecosystem consequences derived from changes in community composition.

White-lipped peccary (*Tayassu pecari*—WLP herein), a species of conservation concern, has been extirpated in 21% of its range over the past century, with reduced abundance and a low to medium probability of long-term survival in 48% of their current range (Altrichter et al. 2012; Richard-Hansen et al. 2014; Fragoso et al. 2020). This has been attributed in some cases to localised population reduction or extirpation due to hunting (Reyna-Hurtado 2009; Reyna-Hurtado et al. 2010), habitat loss, and fragmentation (Keuroghlian et al. 2015). However, recent evidence also suggests that population boom-bust cycles occur across wide regions, and potentially, even biomes (Richard-Hansen et al. 2014; Fragoso et al. 2020). Such boom-bust dynamics have been speculatively attributed to epidemic outbreaks and associated rapid mass die-offs, likely tied to WLP’s tendency to form mega-herds, a unique social structure for a Neotropical mammal (Fragoso 2004).

White-lipped peccary herds are known to reach up to 300 individuals—a collective biomass comparable to that of three African bull elephants roaming throughout the system—and in some cases up to 1000 individuals (Reyna-Hurtado et al. 2016). The herding behaviour likely benefits individuals through improved foraging and defense (see Kiltie and Terborgh 1983). It generates profound impacts on the surrounding environment by influencing a variety of ecosystem services, including seed dispersal (Beck 2006), nutrient recycling, the creation of key microhabitats for other species (Beck et al. 2010), and the top-down control of seedling survival (Silman et al. 2003; Terborgh 2012; Rosin et al. 2017). As such, WLP are known to be one of Amazonia’s key ecosystem engineers or rainforest architects (Thornton et al. 2020).

The reliance of WLP’s on the presence of high value food resources such a fruits and seeds, the availability of which is typically patchy through space and time (Altrichter et al. 2001; Reyna-Hurtado et al. 2012), is thought to render them more susceptible to habitat disturbance than the more omnivorous congener, the collared peccary (*Pecari tajacu*) (Thornton et al. 2020). Collared peccaries show marked dietary overlap with WLP’s and occur sympatrically throughout much of their range (Desbiez et al. 2009). Niche partitioning occurs spatially, whereby collared peccaries occupy sites less frequently when WLP’s are present (Ferreguetti et al. 2018), and temporally, where each may use different diel periods (Hofmann et al. 2016). The reason for this separation could be due to WLP outperforming collared peccaries at acquiring resources, but also directly through interspecific aggression, as WLP kill infant collared peccaries (Carrillo and Fuller 2021). As such, the extirpation of WLP should result in changes in distribution, habitat use, or activity times of collared peccaries.

The absence of WLP may also elicit changes in other frugivorous and herbivorous species due to changes in the availability of nutritional resources previously consumed or destroyed (e.g., trampled) by the mega-herds. WLP’s, where studied, show dietary overlap with other members of the frugivore community, including larger species such as tapir and brocket deer (Camargo-Sanabria and Mendoza 2016). Brocket deer (genus *Mazama*) show a substantial proportion of fruit in their diet (Bodmer 1990) and likely compete with other frugivores for resources, particularly for smaller sized fruits (1-2 cm; Gayot et al. 2004). White-lipped peccaries are also thought to cause the highest non-trophic (non-consumptive) vertebrate damage to the seedling layer in Amazonian systems (Rosin and Poulsen 2016). Their extirpation would likely change resource availability for non-competing herbivores, and as such, alter the composition and longer-term resource availability of the forest understory (Souza et al. 2022).

The presence or absence of WLP herds should logically affect predators that depend on this species as a focal resource. In particular, jaguar (*Panthera onca*) in the Western Amazon have a diet that depends heavily on WLP (Flores-Turdera et al. 2021), and so may respond negatively to WLP extirpation. Puma, on the other hand, a large sympatric felid with similar ecological requirements to jaguar (Foster et al. 2013), exhibit a wider diet breadth, and are not commonly associated with WLP (Flores-Turdera et al. 2021). Clearly, it is important to characterise the role WLP play in impacting the wider terrestrial vertebrate community (Mandujano and Reyna-Hurtado 2019), or how their presence/absence within a system may be compensated by the broader community of species with which they co-occur.

A second putative pathway by which WLP could alter community structure relates to indirect effects mediated by jaguar presence. As WLP represent the principal jaguar food source where species are sympatric (Flores-Turdera et al. 2021), jaguar may switch to prey upon less-preferred species. Alternatively, if jaguars decrease/move away due the absence of WLP, other species may benefit by a reduction of jaguar predation (Estes et al. 2011; Ripple et al. 2014) or interspecific competition (Moreno et al. 2006). These pathways are not necessarily mutually exclusive. Density compensation could be detected as either a demographic response to WLP absence or ecological release manifested by a shift in habitat utilization. However, so far little is known about the behavioral interaction between WLP and other mammals utilizing the same space and dietary items.

To explore the implications of a single species disappearance on the wider vertebrate community composition, we take advantage of a fortuitous natural experiment using camera traps to quantify habitat-use prior to (2006–2007) and post (2019) a widespread population crash in WLP, which occurred around 2010 (Fragoso et al. 2020). To achieve this, we use a temporally replicated camera trapping dataset to examine the effects of WLP disappearance on the terrestrial, medium-large vertebrate community structure within the Los Amigos Conservation Concession (LACC).

Specifically, we explore evidence for changes in vertebrate community habitat-use from pre to post crash periods at LACC, whilst controlling for other factors likely to influence mammal habitat-use. We first confirm the decline in WLP capture rates using a single-species model. We then explore evidence for potential density compensation using a multispecies model to characterise community-wide changes in habitat-use and species rank abundances between the pre- and post-periods. We focus on the response of jaguar (as the largest apex predator in this system), puma (as a smaller apex predator), and the sympatric collared peccary. Finally, we explore the evidence for trophic release through the comparison of community-weighted mean traits (body size, feeding guild and nocturnality) between the pre- and post- crash periods. Changes in the community weighted means between foraging guilds could represent changes consistent with trophic release.

## Methods

### Study site

**Fig. 1:**
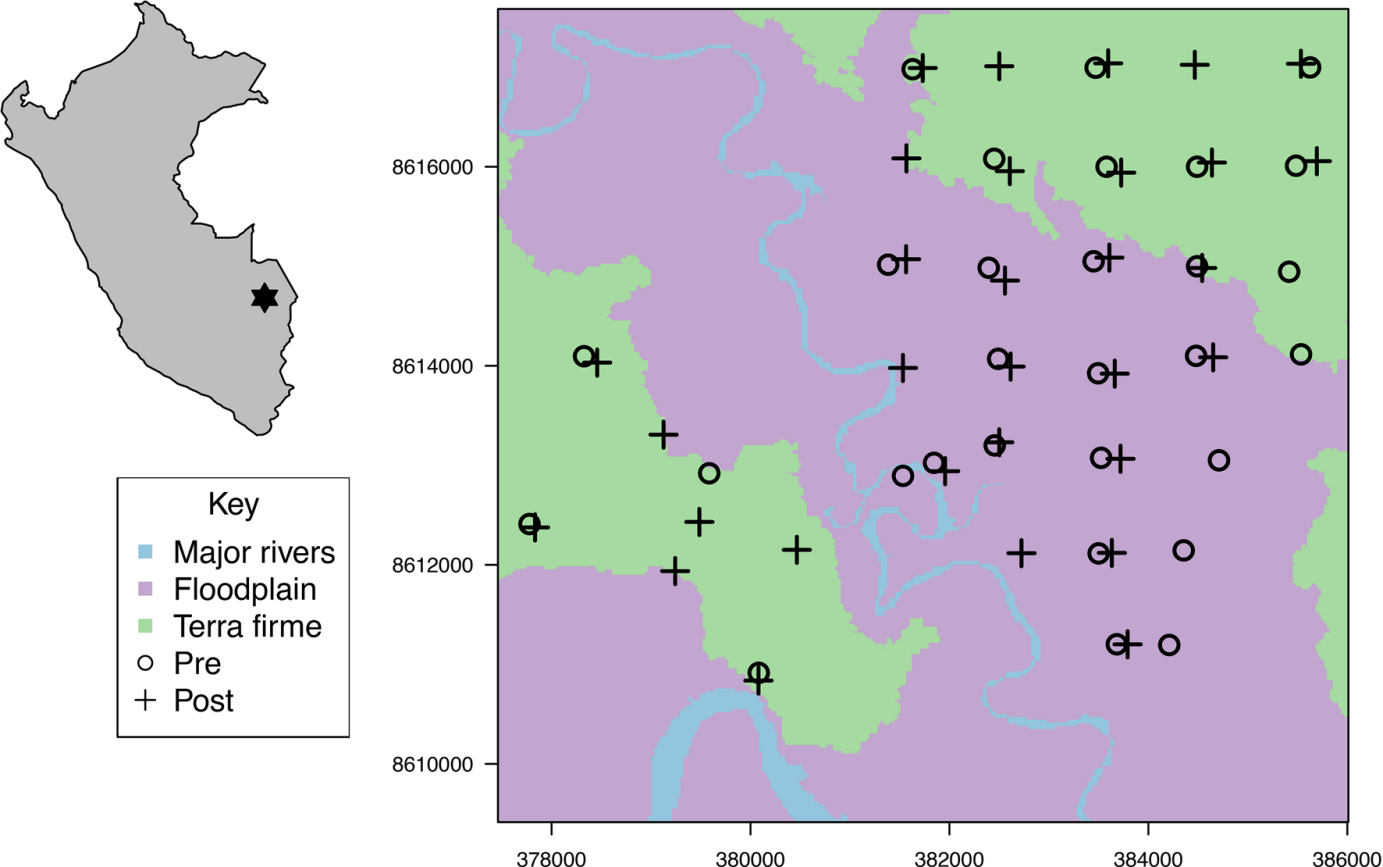
Inset = location of Los Amigos Conservation Concession (LACC) and Los Amigos Biological Station (LABS) in Peru; Main Figure = the location of survey stations and major habitat types in the study area, axis tick-marks represent UTM coordinates (19S) in meters

The study took place within the Los Amigos Conservation Concession (LACC) and the Los Amigos Biological Station (LABS) in Madre de Dios Department in Southeastern Peru (see Fig. 1). The 146,000 ha LACC (12°34′17″S, 70°04′29″W) was established in 2001 and the 453 ha LABS (12°34′0″S 70°05′57″W), a private reserve, was established in 2000. Both protected areas are managed by the Peruvian NGO Conservación Amazónica (ACCA). The two protected areas act as a buffer zone for Manu National Park and for the Madre de Dios Territorial Reserve and contribute to the biological corridor that connects Manu National Park and Tambopata National Reserve. Marco Antonio Texi Valencia logging concession (MATV, 12°30′29.4″S, 70°13′41.6″W) covers 7442 ha and was granted by the government in 2002. MATV is located west of LACC. Habitats include a mixture of *terra firme* (well-drained uplands not subjected to seasonal flooding)*,* floodplain and riverside forests, and palm swamps dominated by *Maurita flexuosa,* and bamboo patches (*Guadua sp. and Olyra sp.;* Guzmán and Stevenson 2008). The area has two predominant seasons: the dry season, from June to September, and the wet season from November to February (Pitman 2010). The mean annual precipitation ranges between 2700 and 3000 mm, and the mean annual temperature ranges between 21 and 26 °C. The habitat diversity in the study region results in a highly diverse community of terrestrial medium and large-bodied mammals; 31 species have been recorded in the study site (Tobler et al. 2008). This survey was carried out in *terra firme* and floodplain forests, which covers the largest land area in the region (Pitman et al. 1999).

### Camera trapping

#### Pre-population crash

Tobler and colleagues deployed un-baited, on-trail, paired camera traps in 40 locations during 2006 and 2007 [Deer Cam (NonTipical Inc.) 35 mm film passive infrared cameras]—22 in LACC, 2 in LABS, and 16 in three logging concessions (including MATV), with a mix of 1 km and 2 km spacing deployed 50 cm from the ground with a 5-min delay between pictures (see Tobler et al. 2008). The survey covered ~ 50 km^2^ of *terra firme* and floodplain forests within the dry season (sampling occurred between 15th of August–17th of October in 2006 and the 2nd of September–7th of November in 2007). This original suite of camera trap deployment locations was reduced to only those possible to repeat in 2019 (see details below), resulting in a pre-crash sample size of 28 deployment locations in 2006 and 28 deployment locations in 2007 covering ~ 48 km^2^ (see Fig. 1). Thirteen deployment locations were in floodplain and 15 were in *terra firme* habitats.

#### Post-population crash

In 2019 we replicated the camera trap grid surveyed in 2006–2007 (Tobler et al. 2008) with the aim to detect changes in the medium and large-bodied terrestrial vertebrate community related to WLP disappearance. However, because of the current inaccessibility to two of the three logging concessions previously surveyed (Madefol S.A.C and Empefomsba S.A.C), these sites were excluded from both the pre and post analysis. This left thirty-one camera locations [24 in LACC, 1 in LABS, and 6 in MATV] were deployed in the dry season from 19 June to 27 October 2019 (4 months) covering ~ 49 km^2^. Twenty-two of the deployed stations matched the original locations surveyed, the remaining stations were within the same focal survey area (< 2 km from existing locations; see Fig. 1). Fourteen deployment locations were within floodplain and 17 were within *terra firme* habitats. All camera trap stations were deployed on-trail, unbaited, and with an approximate spacing of 1 km between stations. As in Tobler et al. (2008), each deployment location consisted of a pair of camera traps, one on each side of the trail. Two camera models were used: Bushnell Trophy Cam HD aggressor 119776c 20MP Low Glow and Browning Trail Camera SPEC OPS BTC-6HDX 16MP. Twenty camera trap stations consisted of one Bushnell camera and one Browning camera, and the remaining 11 camera trap stations consisted of two Bushnell cameras. All cameras were installed at a height of ~ 50 cm from the floor and set to record three photos with a 30 s rest period upon trigger by motion.

To increase the comparability of the 2019 data with that collected in 2006 and 2007, we split the 2019 deployments into two, eight-week sessions to match the survey effort from each pre-crash deployment period. After the split, the 2019 camera deployments were, on average, 63.7 days in duration (min = 54; max = 74) this is comparable to an average of 62.5 during the pre-crash monitoring (min = 40; max = 67). To increase the comparability of the two datasets (which used differing rest periods between detections) and to increase the independence the data points, the raw camera detection data was aggregated into ‘independent detections’ by collapsing the detection events of a given species at a given deployment location that occurred within 30 min of a previous detection into a single event (Rovero and Zimmermann 2016).

### Detection data management

All terrestrial species which could be reliably identified to species-level, > 1 kg and with > 10 independent detections were included in the analysis (Appendix A). Nine-banded and long-nosed armadillos were grouped together (*Dasypus sp.*) due to difficulties in species discrimination from camera trap images.

Performing longitudinal comparisons with changing technology has potential limitations. To get around this issue we use three different approaches: (1) Rather than focus on absolute changes in species detection rates between pre and post, we focus on relative changes in species ranks. (2) We assess within species relative changes within strata of interest (e.g., the proportion of detections occurring within floodplain vs. *terra firme* forests). (3) We assess how species traits are influenced by the change from pre to post disappearance (e.g., if modern cameras are more effective at detecting small body sized animals, the effect of pre-post disappearance should be correlated with an increase in the proportion of small-bodied animal detections).

We assumed that our response variable—the number of independent detection events per deployment —was an index of ‘habitat-use’ by each species (e.g., Rovero et al. 2014; Cusack et al. 2015) and analyzed the data using two Bayesian models (described below). Although the index of habitat-use does not directly account for imperfect detection (an animal using the location, but not detected), the occupancy modelling framework often proposed to address imperfect detection is also susceptible to bias related to animal movement behavior in camera trap surveys (e.g., Neilson et al. 2018). We focus interpretation on within-species shifts in habitat-use rather than direct comparison of detection rates between species, which will be driven by between species variation in traits (e.g., home range size, mobility, and body size; Devarajan et al. 2020).

### Single species model

To examine the general trends in WLP detection rate through time we fitted a single species Bayesian generalized linear mixed models using brms R package (Bürkner 2017) with a log link (Poisson). We included a two-level factor related to whether the deployments occurred during the pre (2006–2007) or post (2019) crash period, and an interaction with a two-level factor relating to habitat type (*terra firme* or floodplain) to explore habitat-specific variation in use—WLP have been known to prefer floodplain areas close to rivers, where palm stands are abundant as a food resource (Keuroghlian and Eaton 2008; Keuroghlian et al. 2009; Mandujano and Reyna-Hurtado 2019). We included a fixed effect representing the log number of camera nights at each station to account for inter-deployment location variation in survey effort, and deployment location as a random intercept to account for non-independence between repeated observations associated at the same location. We used four chains each with 2000 iterations, with the first 1000 samples discarded as burn-in, resulting in 1000 samples per chain.

### Community model

To assess the wider community response to WLP disappearance, we implemented a joint species distribution model which allows us to simultaneously control for the effect of covariates and the role of traits in driving species-level responses. We used the Bayesian ‘Hierarchical Modelling of Species Communities’ (HMSC) package v3.0 (Tikhonov et al. 2020) within the R statistical environment. The response term in our models was a two-dimensional ‘station_time’ by species matrix, where each row specified the number of independent detection events within each deployment for each species. In addition to the two-level factor representing population status (pre/post-crash), habitat (*terra firme*/floodplain), and their interaction term described above, we also control for two additional covariates which could influence species habitat-use: distance to nearest community, as a proxy for human disturbance; and distance to the nearest navigable river as a proxy for human accessibility. Evidence for overt changes in temperature and rainfall regime we absent through the study period (Appendix B), and forest degradation (e.g., logging) between the two survey periods was also negligible as the area is protected (Appendix C). We included the log of the number of camera nights at each deployment location as a fixed effect. We used a Bayesian framework with Markov Chain Monte Carlo (MCMC) to estimate the model parameters. Species counts were assumed to follow a Poisson distribution, and species responses to the predictors were assumed to follow a multivariate Gaussian distribution. We used the default, non-informative priors (Tikhonov et al. 2020). The final model was fit with four MCMC chains, each composed of 505,000 iterations with a thinning interval of 500 and a burn in length of 5000, resulting in 1000 samples per chain. Parameters were confirmed to have converged and mixed well through visual inspection of trace plots, examination of effective sample size and potential scale reduction factor. We used pseudo-R2 as a measure of model fit, calculated as the squared Spearman correlation between observed and predicted values, times the sign of the correlation (Ovaskainen and Abrego 2020).

We also calculated the proportion of explained variance that was attributable to each of the fixed effects in the model and used this as a measure of relative importance. We used model-estimated parameters to predict species-specific habitat-use under ‘pre’ and ‘post’ crash scenarios at average community distance and distance from navigable rivers. We defined species for whom credibility intervals between ‘pre’ and ‘post’ crash periods do not overlap as a suite of species which have the potential to be influenced by WLP disappearance. Finally, we explored if the magnitude of change between ‘pre’ and ‘post’ crash sampling periods is related to species traits extracted from the EltonTraits database (Wilman et al. 2014): (i) nocturnality—a two level factor for ‘active at night’, or ‘not active at night’ (exclusively diurnal and/or crepuscular); (ii) body mass (kg) and (iii) dietary guild—a five level factor reflecting the majority diet: Frugivore/Nectorivore; Insectivore; Omnivore; Plant/Seed; or Carnivore/Scavenger. We interpret model coefficients where credibility intervals do not include zero as significant.

## Results

### White-lipped peccaries

**Fig. 2:**
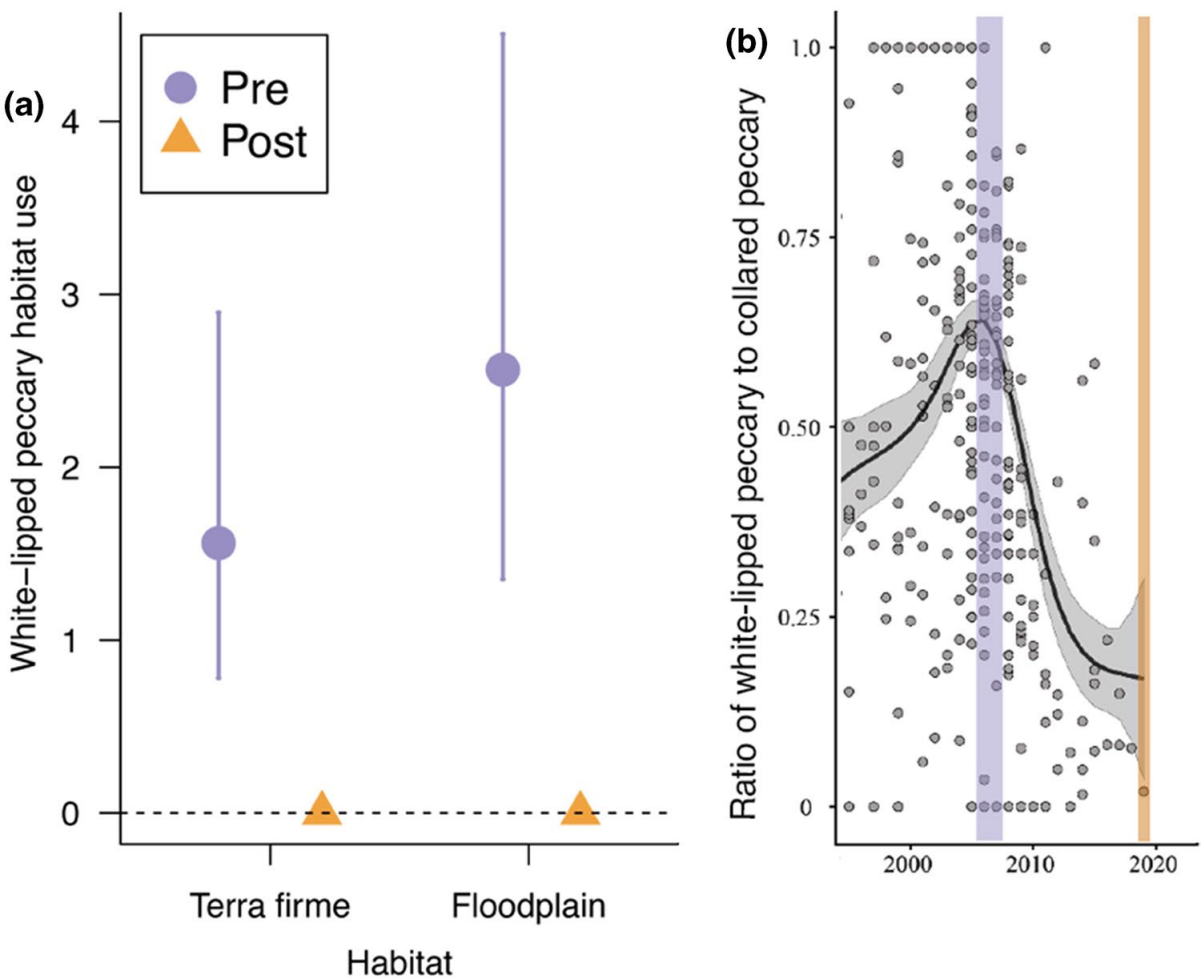
**A** Mean and 95% credible intervals of the predicted habitat use for WLP in the pre-crash (2005/2006, purple and circles) and post-crash (2019, orange and triangles) periods, and between two major habitat types; *terra firma* and floodplain. **B** Focal camera trapping periods overlayed on the hunting off-take data from the region—adapted from *Mysterious disappearances of a large mammal in Neotropical forests* (Fragoso et al. 2020)

Comparison of the WLP capture rates between the ‘pre’ (2006/2007) and ‘post-crash’ (2019) surveys confirmed that WLP habitat-use declined from 1.56 detection events per 63 camera-trap nights (CI 0.78–2.90) in *terra firme* habitat and 2.56 detection events per 63 camera-traps nights in floodplain habitat (CI 1.35–4.51) to zero in both habitats (Fig. 2A). These patterns are consistent with the proposed crash event periods described by Fragoso et al. (2020) based on the ratio of white-lipped peccary to collared peccary in the southwestern Amazon (recreated in Fig. 2B). Pre-crash, WLP habitat-use was on average higher in floodplain habitat than *terra firme* (2.56 vs. 1.56, respectively), although the 95% credibility intervals for the floodplain habitat co-efficient encompassed zero (mean = 0.46; CI − 0.43–1.31).

### Community response to WLP absence

Of the variance explained by our community-level model, pre-post survey was the most important predictor, accounting for 27.4% of the variation explained, followed by the interaction between habitat and pre-post survey (13.1%), and habitat type (10%, see Appendix D). Community distance explained slightly more variation than river distance (12.2% vs. 10.4%, respectively).

**Fig. 3:**
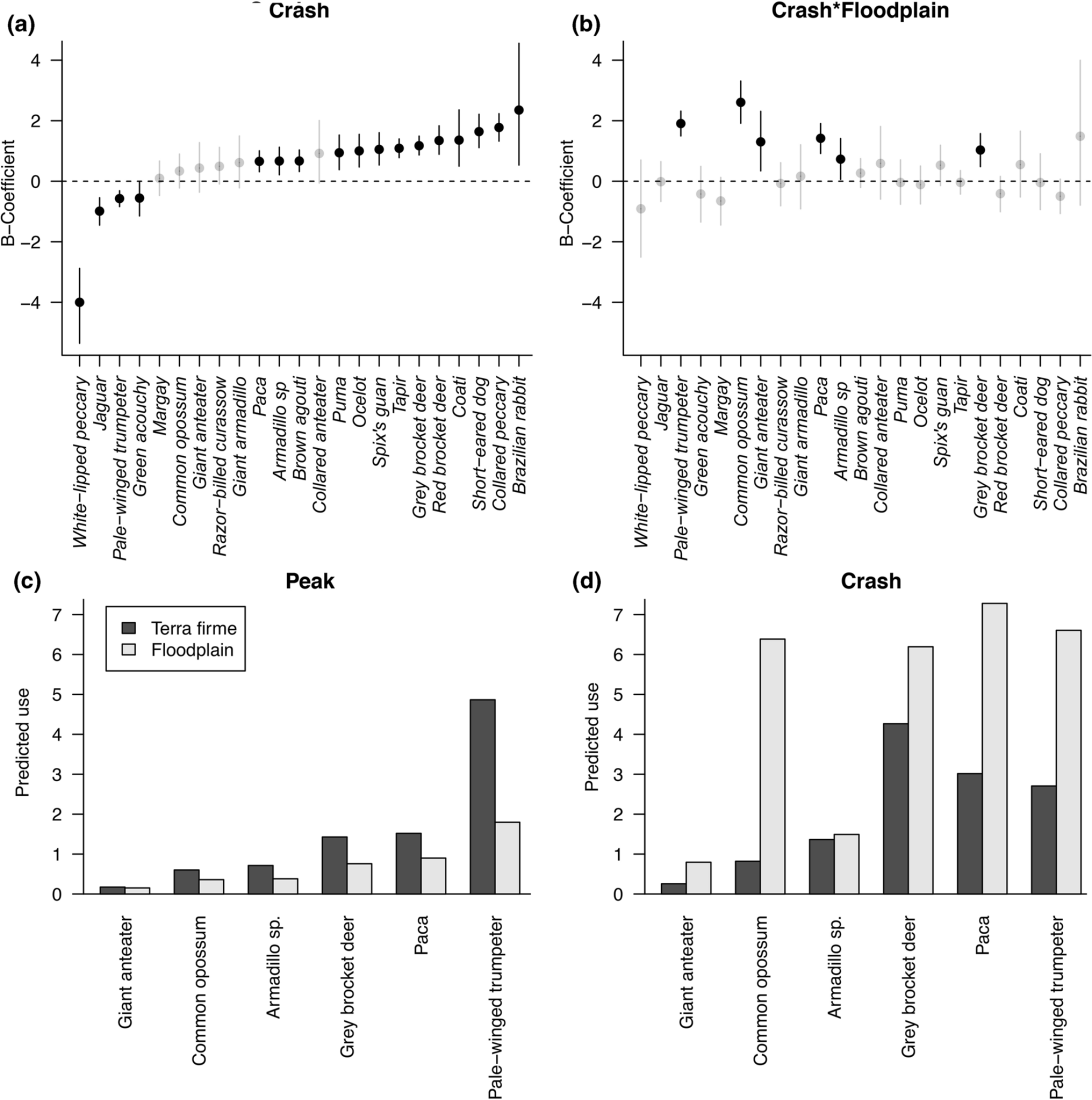
**A** The mean effect size and 95% credibility intervals for the ‘post’ crash co-efficient (relative to ‘pre’ crash) species-level habitat use; **B** mean effect size and 95% credibility interval for the interaction between ‘crash’ period and floodplain habitat species-level habitat use; **C** and **D** Predicted habitat use in *terra firme* and floodplain for the species where there is evidence for an interaction with sampling period (**C** pre; **D** post)

During the WLP crash period, 19 of 22 terrestrial vertebrate species displayed an increase in capture rate between survey periods—13 of them significantly (see Fig. 3A; Appendix E). Just three of the 22 species displayed a reduced capture rate following the absence of WLP (Fig. 3A).

Six species displayed significant increases in use of floodplain habitat following the population crash of WLP (i.e., species that were previously found in greater frequency in *terra firme* when WLP were abundant and encountered more in floodplain following WLP absence; Fig. 3B). These were *Myrmecophaga tridactyla* (giant anteater), *Didelphis marsupialis* (common opossum), *Dasypus* armadillos, *Mazama gouazoubira (*gray brocket deer), *Cuniculus paca* (paca), and *Psophia leucoptera* (pale-winged trumpeter) (Fig. 3C and D).

**Fig. 4:**
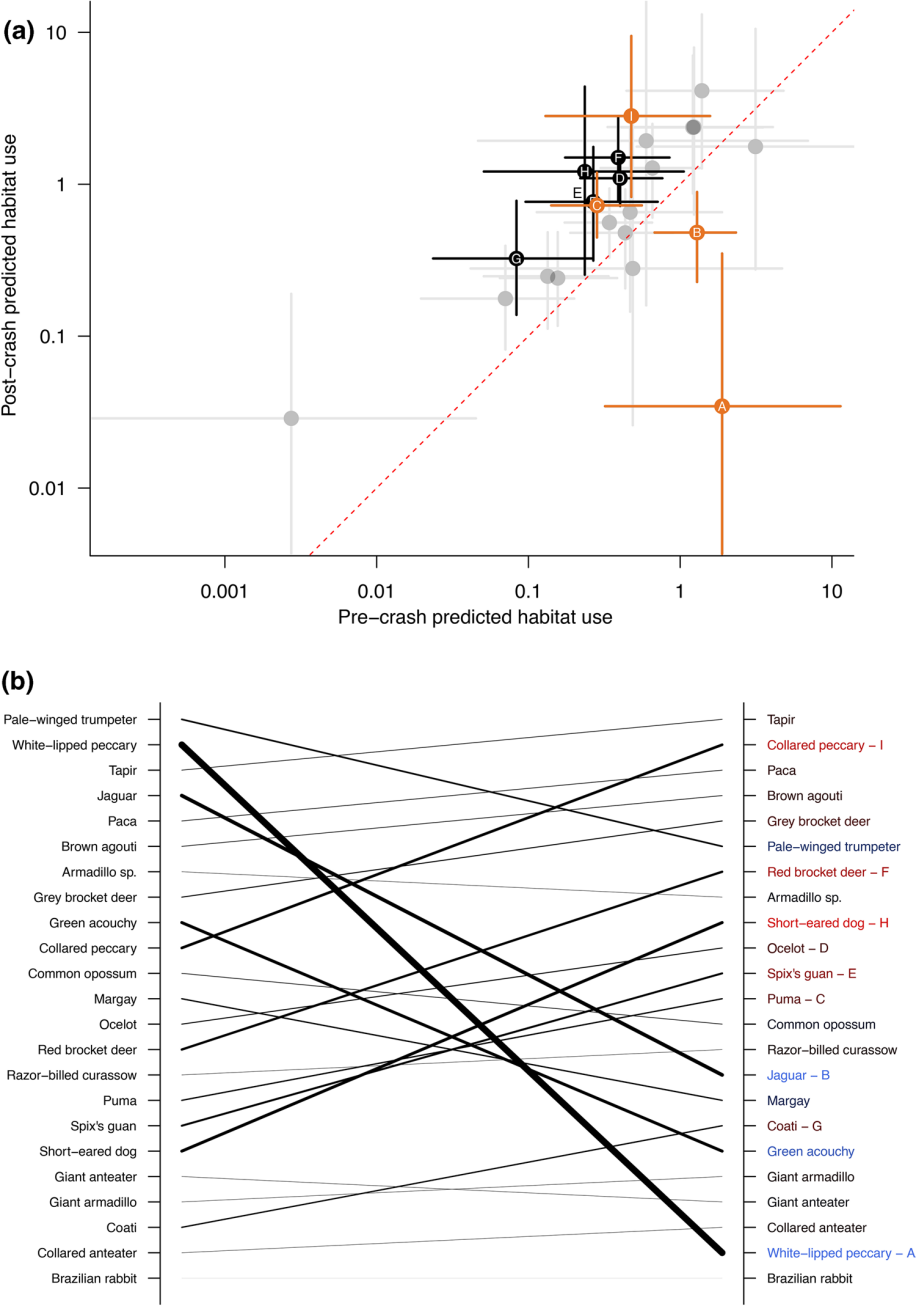
**A** Predicted changes in habitat use from pre- (x-axis) and post- (y-axis) crash periods, where significant changes are indicated by species whose 95% credibility intervals do not overlap the dashed line (after controlling for the fixed effects). Where species codes = white-lipped peccary (**A**), jaguar (**B**), puma (**C**), ocelot (**D**), razor-billed curassow (**E**), red-brocket deer (**F**), South American coati (**G**), short-eared dog (**H**), collared-peccary (**I**). Species above the line represent increases, below the line, decreases. Bold species crosshairs represent significant increase/decline; jaguar, WLP, puma and collared peccary are highlighted in orange. **B** Species ranked by predicted habitat use during pre- (left) and post- (right) crash. Asterixis denote species with significant changes as defined in (**A**). Colours and width of lines denote the magnitude of rank changes, where red = increased rank, blue = decreased rank, and black suggests no major change. The thickness of the line relates to the respective degree of shift in rank (i.e., thicker lines represent a greater degree of change in rank)

The effects of crash period parameter translated into marked changes in relative capture rate and community rank order for eight species (Fig. 4A and B). Seven species—*Puma concolor* (puma), *Leopardus pardalis* (ocelot), *Mitu tuberosum* (razor-billed curassow), *Mazama americana* (red-brocket deer), *Nausa nausa* (South American coati), *Atelocynus microtis (*short-eared dog), and *Pecari tajacu* (collared peccary)—displayed an increase in their rank ordered position; in addition to WLP only the jaguar showed a major decrease in rank order (Fig. 4B). The greatest changes in species rank in pre- and post- events were of collared peccary (from 10th to 2nd), red-brocket deer (from 14th to 7th), and short-eared dog (18th to 9th), while jaguar fell in rank following the disappearance of WLP, from 4th to 15th (see Fig. 4B). Puma increased in rank from 16th to 12th, shifting to a higher rank than jaguar. In the absence of WLP, tapir became the most frequent, highest ranked order species in the community (from 3rd).

### Other predictors of terrestrial vertebrate distribution

Eight species from the community model displayed significant differences in habitat-use between floodplain and *terra firme*. Razor-billed curassow, ocelot, Spix’s guan, Brazilian tapir and red brocket deer displayed greater encounter rates within floodplain, while pale-winged trumpeter, paca, and *Dasypus* armadillos were detected more frequently in *terra firme* (see Appendix E). Nine species showed significant differences in habitat-use in relation to distance from river: pale-winged trumpeter, giant anteater, razor-billed curassow, puma, ocelot, Brazilian tapir, red brocket deer, and collared peccary all displayed a lower rate of detection with increasing distance from the river. Only green acouchi showed a greater rate of detection farther away from the river. Nine species displayed significantly different encounter rates in relation to the proximity of nearby communities. Paca, Spix’s guan, Brazilian tapir, and both red and gray brocket deer were encountered more frequently farther away from communities. Common opossum, puma, ocelot, and Brazilian rabbit on the other hand, were all detected more frequently in camera stations closer to the neighbouring communities.

### Species-level predictors of compositional change

We found no evidence to support consistent community-level changes in either feeding guild composition or body size between pre- and post- crash periods, and no change in nocturnality either (see Appendix F). Smaller animals were not detected more frequently with modern camera traps (post disappearance) as we might have expected, and species that are active at night did not display higher counts post population crash. The only significant trait response detected was that relatively more diurnal species were encountered with increasing distance from villages (or converse, more nocturnal species are recorded closer to communities; see Appendix F).

## Discussion

Examples of the extirpation of large-bodied species from large contiguous tracts of habitat, in the absence of mechanisms that would directly influence presence of other community members (e.g., hunting and habitat fragmentation), are rare. We recognise that an observational study such as this cannot demonstrate a causal link between WLP disappearance and habitat-use changes in co-occurring species. However, by taking advantage of fortuitous pre- and post-crash surveys, we provide the first temporally and spatially replicated assessment of how population crash events of WLP alters the structure of vertebrate communities—notably in the absence of other overt anthropogenic disturbances. While we detected no change in guild species composition, we detected a marked increase in the camera capture rates of collared peccary, red-brocket deer, and puma, and decrease in jaguar, the implications of which we discuss below.

Relative to the WLP pre-crash period, we detected a significant reduction in encounters of the systems top terrestrial felid predator, the jaguar. Despite the decline in jaguar, we found no clear evidence of a trophic cascade event, as the trait composition of the community post-disappearance was not significantly different from the pre-disturbance community. Rather, our results are more consistent with density compensation—whereby similar sympatric species demographically compensate for the loss of WLP and decline in jaguar. Consistent with this, the most marked change was that of the collared peccary, a WLP sympatric species fulfilling a similar niche space—known not only to be controlled in terms of resource competition, but also directly via WLP killing juvenile collared peccary (Carrillo and Fuller 2021). Collared peccary shifted from 10th in rank habitat-use when WLP’s were abundant, to 2nd when WLP were absent. Although we would expect the extirpation of a high biomass ecosystem engineer, such as WLP, to have ramifications on the survival and composition of common tree species in tropical forests (Silman et al. 2003; Terborgh 2012; Rosin et al. 2017), we found that in the absence of other stressors (e.g. habitat degradation), that such changes may be offset by the density compensation observed by other vertebrates (such as collared peccary and red-brocket deer). This process could also occur in other taxa/systems with marked functional trait redundancy (e.g., Nunes et al. 2021).

Such changes are consistent in support of a direct pathway of density compensation, whereby the terrestrial vertebrate community in the absence of the sympatric WLP herds can take advantage of the additional available resources, boosting their populations. In addition to an overall increase in encounter rate for many species generally, we found evidence of ecological release of the habitat utilization of some species for floodplain over *terra firme* was correlated with the absence of WLP. This suggests that WLP influence the habitat selection of other Amazonian terrestrial vertebrates through direct competition or indirect pathways. It could also reflect species responses to environmental changes, which are also correlated with WLP extirpation, such as climatic changes and resource abundance (e.g. Esquivel‐Muelbert et al. 2019; Neate-Clegg et al. 2021; Stouffer et al. 2021). Given the long interval between survey periods and observational nature of this comparison, it is not possible to tease these mechanisms apart.

In contrast to the increases in habitat-use observed in many species, the system’s apex predator, the jaguar, was the only species that displayed substantial declines in both encounter rate and community rank-abundance. This reduction in jaguar habitat-use, but continued presence, supports the existence of a strong predator–prey relationship and diet preference of WLP by jaguars in the Western Amazon (Flores-Turdera et al. 2021). However, jaguar can evidently avoid local extirpation by consuming other species (Hayward et al. 2016), as is well documented in Central America and other regions of the Amazon (Hernández 2008). The extent to which such changes represent demographic or behavioural responses are as yet unknown (i.e., does reduced habitat-use reflect a demographic decline or behaviour adaption?); and this warrants further investigation.

In addition to puma, one other felid, the ocelot, and a canid, the short-eared dog, each showed increased capture rate and community rank following the disappearance of WLP and reduction in jaguar presence. This suggests that some carnivores also display density compensation in response to the loss of WLP and reduced presence of jaguar (Moreno et al. 2006). The only change in community trait composition we identified was nocturnality and distance to community, with species closer to communities generally being, on average, more nocturnal. This seems to agree with the concept of a defaunation halo-effect radiating outwards due to the hunting affects by communities (Beirne et al. 2019; Scabin and Peres 2021), and a selection for more nocturnal species/behaviours under hunting pressure (Espinosa and Salvador 2017). We also detected that some species displayed a preference for floodplain or *terra firme* forest in agreement with other Amazonian mammal surveys (Salvador et al. 2011; Costa et al. 2018). However, as data collection was only conducted in the dry season; wet season patterns may differ from those described here.

Given that the study site has been protected and patrolled since 2001 and its wildlife is under recovery from historic disturbance (Terborgh 2012), we suggest that hunting is not the principal reason for the precipitous decline and disappearance of WLP at LACC and LABS, nor the corresponding effects observed in other species. The 146,000-ha concession has challenges with illegal logging on its peripheries and gold mining on the main river (outside the protected areas to the south), with the potential for low-level hunting for subsistence, or trade, close to the bordering logging concessions. However, the concession’s core is remote Amazonian protected wilderness. If hunting was the principal impact associated with local extirpation, then we would expect to see a similar signal in other species also targeted by hunters. In contrast, we observed that in the absence of WLP, other hunted herbivores, such as deer, tapir, collared peccary, paca (see Scullion et al. 2021), displayed significant increases in habitat-use. Consequently, hunting is an unlikely mechanistic candidate for the sudden major collapse in WLP habitat-use observed at this study site. This also suggests that the principal driver of changes observed in this landscape is unlikely to be the pet trade (e.g., Sinovas et al. 2017; Fukushima et al. 2020).

The potential for a major WLP migration away from the focal region, although possible, seems implausible given the sudden and coordinated declines in WLP numbers also observed in other locations in the Amazon (Fragoso 2004; Richard-Hansen et al. 2014; Fragoso et al. 2020). They also coincide with opportunistic field observations of parallel crashes in adjacent protected areas around the same time (see Appendix G). Although still to be conclusively proven, there is evidence to suggest that peccaries are susceptible to various forms of swine flus and diseases (de Castro et al. 2014; D’arc et al. 2020), likely due to their distant, albeit common ancestry with pigs around a million years ago (de Castro et al. 2014). Regardless of cause, similar large-scale, synchronous declines have not been documented in collared peccary populations. This does not necessarily mean that they could not be carriers of some form of swine disease, rather they might be more resilient or unaffected themselves—either directly physiologically, or in relation to their natural history associated with smaller group sizes.

It is important to note that other studies, albeit typically conducted at longer time scales of > 20 years, have concluded that compositional changes in vertebrate communities, like those described here, could be due to the cumulative effects of climate change (Esquivel‐Muelbert et al. 2019; Neate-Clegg et al. 2021; Stouffer et al. 2021), as opposed to the loss of an ecosystem engineer, like WLP. It is difficult to tease apart these two processes in an observational pre-post comparison. However, we have shown that overt changes in rainfall and temperature have not occurred within the region between the two survey periods (Appendix B), and highlight research which suggests that WLP extirpation could have profound effects on understory plant composition and resource availability (Rosin et al. 2017).

An additional challenge with observational pre-post comparisons is that ‘experimental’ effects are confounded with changing hardware. We are comparing data from older film and flash camera trap models (as used in the 2006–2007 surveys) with modern infra-red digital camera traps (used in the 2019 surveys). The technological improvements in camera traps led us to predict that we would observe improvements in the detection probability of smaller species, and perhaps more nocturnal animals due to enhanced nocturnal sensing capabilities and potential reduced avoidance by wildlife associated with old incandescent flashes (Schipper 2007). Although we did notice a general increase in overall capture rates between pre- and post- survey periods, we found no strong support for such changes to be related to nocturnality or body size traits. This leads us to believe that although our data might detect general overall improvements in relation to capture rate by modern cameras, these are not specific to subsets of the community and are therefore unlikely to be responsible for any observed shifts in community structure or ranks that we have identified due to the population crash of WLP.

The continued monitoring of crash events and changes within the wider terrestrial vertebrate community is key to our deeper understanding of potential longer-term downstream impacts. Anecdotal reports from LACC in the past 24 months have observed small WLP groups (10–30 individuals) returning; and have been confirmed on at least two camera trap locations from LACC. In terms of the cycles proposed by (Fragoso et al. 2020) we might predict that this could be the early stages of population recovery, where WLP once again become a dominant species at LACC in the future. If so, our data leads us to predict that the relative encounter rates of collared peccary, red-brocket deer, and puma (along with other species) will decrease, and that jaguar habitat-use will increase with WLP recovery.

Understanding the response of communities to the disappearance of individual species can give insights into the mechanisms regulating community composition and the implications for ecological processes. Here, we conclude that the extirpation of WLP, be it related to localized hunting extirpation events, mass die-offs, or herd migrations (Endo et al. 2010), leads to marked shifts to the Amazonian terrestrial vertebrate community structure through density compensation and ecological release. Despite this, there was no detectable shift in foraging guild structure within this system, suggesting that the loss of WLP and decline in jaguar were offset by species occupying similar niches (e.g., collared peccary and puma). The density compensation detected here may only be possible in high-diversity landscapes where sympatric species show redundancy in trait space. Similar changes in lower productivity landscapes would presumably have a greater effect on trait composition. Continued monitoring of crash events and changes within the wider terrestrial vertebrate community, and crucially, how such changes influence ecosystem processes and services, should be a focus of future research.

## Supplementary Information

Below is the link to the electronic supplementary material.

Supplementary file1 (DOCX 353 KB)

## Data Availability

Available from author CB upon reasonable request.

## Code availability

Available from author CB upon reasonable request.

## Declarations

### Research approval

Research approval was given by Gobierno Regional de Madre de Dios (GOREMAD) with the permit N# 043–2019-GOREMAD-GRRNYGMA-DRFFS/AF/brcr).
