# Supplementary material for: Disappearance of an ecosystem engineer, the white-lipped peccary (Tayassu pecari), leads to density compensation and ecological release
Source: Oecologia. 2022 Aug 13;199(4):937–49. doi: 10.1007/s00442-022-05233-5 (PMC9464176; doi:10.1007/s00442-022-05233-5)
Supplement: Supplementary file 1 — Supplementary file1 (DOCX 353 KB) [file 442_2022_5233_MOESM1_ESM.docx]

**Appendices**

**Appendix A:** Capture rate summary table

| **Common name** | **Species** | **PreCR** | **PostCR** | **PreOc** | **PostOc** | **Strata** | **Nocturnal** | **Mass_g** | **Diet** |
| --- | --- | --- | --- | --- | --- | --- | --- | --- | --- |
| Armadillo sp. | *Dasypus sp.* | 0.89 | 2.37 | 0.3 | 0.5 | G | 1 | 3500 | Invertebrate |
| Brazilian rabbit | *Sylvilagus brasiliensis* | 0 | 0.66 | 0 | 0.06 | G | 1 | 950 | PlantSeed |
| Brown agouti | *Dasyprocta punctata* | 2.09 | 4.19 | 0.45 | 0.66 | G | 0 | 2675 | FruiNect |
| Bush dog | *Speothos venaticus* | 0 | 0.03 | 0 | 0.02 | G | 0 | 6000 | VertFishScav |
| Capybara | *Hydrochoerus hydrochaeris* | 0 | 0.03 | 0 | 0.02 | G | 0 | 48145 | PlantSeed |
| Coati | *Nasua nasua* | 0.09 | 0.63 | 0.05 | 0.29 | S | 0 | 3794 | FruiNect |
| Collared anteater | *Tamandua tetradactyla* | 0.09 | 0.3 | 0.05 | 0.18 | S | 1 | 5515 | Invertebrate |
| Collared peccary | *Pecari tajacu* | 1.14 | 6.39 | 0.45 | 0.76 | G | 1 | 21267 | PlantSeed |
| Common opossum | *Didelphis marsupialis* | 0.74 | 8.89 | 0.34 | 0.6 | S | 1 | 1091 | Omnivore |
| Crab-eating raccoon | *Procyon cancrivorus* | 0.14 | 0.1 | 0.09 | 0.02 | G | 1 | 6950 | Omnivore |
| Giant anteater | *Myrmecophaga tridactyla* | 0.26 | 0.88 | 0.14 | 0.27 | G | 1 | 22333 | Invertebrate |
| Giant armadillo | *Priodontes maximus* | 0.2 | 0.4 | 0.09 | 0.18 | G | 1 | 45360 | Invertebrate |
| Green acouchy | *Myoprocta pratti* | 1.2 | 0.88 | 0.25 | 0.16 | G | 0 | 967 | FruiNect |
| Grey brocket deer | *Mazama nemorivaga* | 1.63 | 8.59 | 0.23 | 0.73 | G | 0 | 17000 | PlantSeed |
| Jaguar | *Panthera onca* | 2.09 | 0.76 | 0.57 | 0.31 | G | 1 | 100000 | VertFishScav |
| Jaguarundi | *Puma yagouaroundi* | 0.06 | 0.03 | 0.04 | 0.02 | G | 0 | 6875 | VertFishScav |
| Margay | *Leopardus wiedii* | 0.83 | 0.68 | 0.36 | 0.32 | S | 1 | 3250 | VertFishScav |
| Ocelot | *Leopardus pardalis* | 1.2 | 2.55 | 0.41 | 0.5 | G | 1 | 11900 | VertFishScav |
| Paca | *Cuniculus paca* | 1.86 | 7.73 | 0.43 | 0.82 | G | 1 | 8173 | PlantSeed |
| Pacarana | *Dinomys branickii* | 0 | 0.03 | 0 | 0.02 | G | 1 | 12500 | PlantSeed |
| Pale-winged trumpeter | *Psophia leucoptera* | 4.72 | 7.6 | 0.7 | 0.74 | G | 0 | 1316 | FruiNect |
| Puma | *Puma concolor* | 0.69 | 1.59 | 0.27 | 0.37 | G | 1 | 51600 | VertFishScav |
| Razor-billed curassow | *Mitu tuberosum* | 0.94 | 1.41 | 0.38 | 0.4 | G | 0 | 2769 | FruiNect |
| Red brocket deer | *Mazama americana* | 1.17 | 3.66 | 0.38 | 0.63 | G | 1 | 22800 | PlantSeed |
| Short-eared dog | *Atelocynus microtis* | 0.31 | 2.17 | 0.16 | 0.4 | G | 1 | 7750 | VertFishScav |
| Southern naked-tailed armadillo | *Cabassous unicinctus* | 0 | 0.03 | 0 | 0.02 | G | 1 | 4800 | Invertebrate |
| Spix's guan | *Penelope jacquacu* | 0.69 | 3.21 | 0.27 | 0.68 | S | 0 | 1488 | FruiNect |
| Tapir | *Tapirus terrestris* | 3.55 | 10.48 | 0.7 | 0.92 | G | 1 | 207501 | PlantSeed |
| White-lipped peccary | *Tayassu pecari* | 5.63 | 0 | 0.77 | 0 | G | 1 | 32234 | PlantSeed |

**Appendix B:** Temporal patterns in temperature and rainfall between the two survey periods shows the lack of overt changes in temperature (upper) and rainfall (lower) within the study region and period.

Data derived from Copernicus Climate Change Service (C3S) (2017): ERA5: Fifth generation of ECMWF atmospheric reanalyses of the global climate. Copernicus Climate Change Service Climate Data Store (CDS) <https://cds.climate.copernicus.eu/cdsapp#!/home>


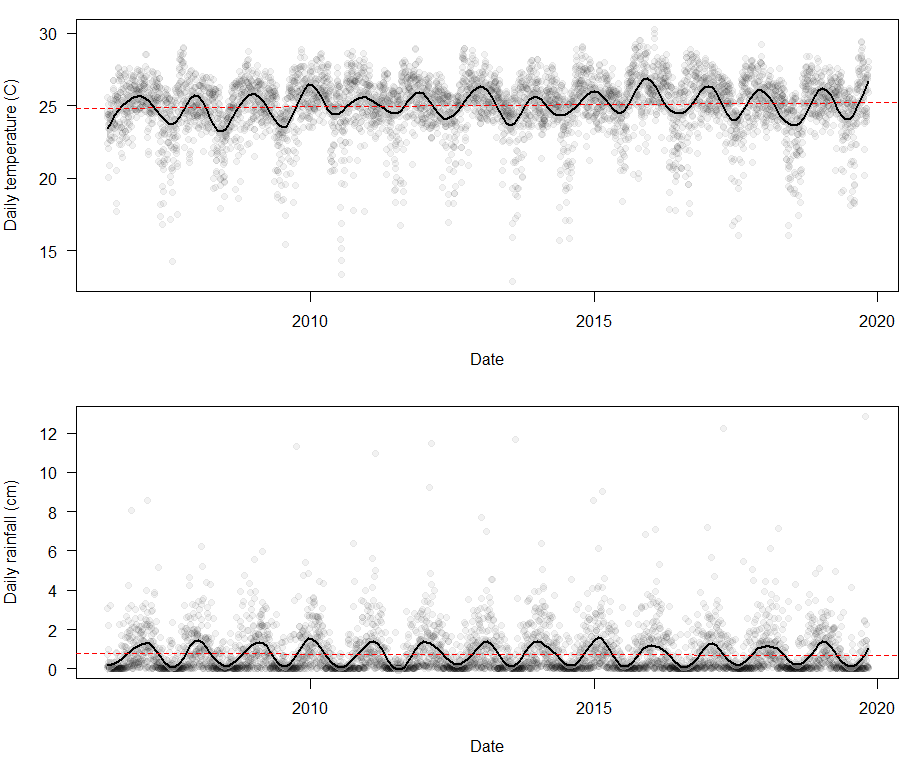


**Appendix C:** Forest cover loss within and surrounding the Los Amigos Conservation Concession (2000-2019).

The following image shows canopy cover loss (green) within and immediately around the Los Amigos Conservation Concession (black border), camera trap stations (Black points). It is important to note that loss can arise due to anthropogenic and natural causes. Within LACC, between 2000-2019 this represents a canopy cover loss of 0.12%.

Data derived from Hansen, M. C., P. V. Potapov, R. Moore, M. Hancher, S. A. Turubanova, A. Tyukavina, D. Thau, S. V. Stehman, S. J. Goetz, T. R. Loveland, A. Kommareddy, A. Egorov, L. Chini, C. O. Justice, and J. R. G. Townshend. 2013. “High-Resolution Global Maps of 21st-Century Forest Cover Change.” Science 342 (15 November): 850–53. Data available on-line at: <https://earthenginepartners.appspot.com/science-2013-global-forest>


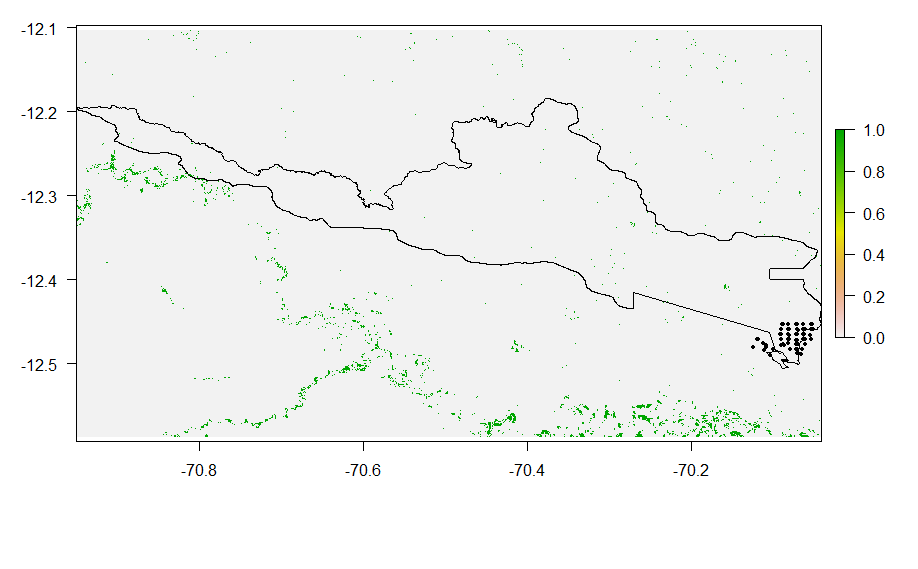


**Appendix D:** Variance explained by the multi-species model

*Species-level ‘total’ pseudo-R^2^*

**
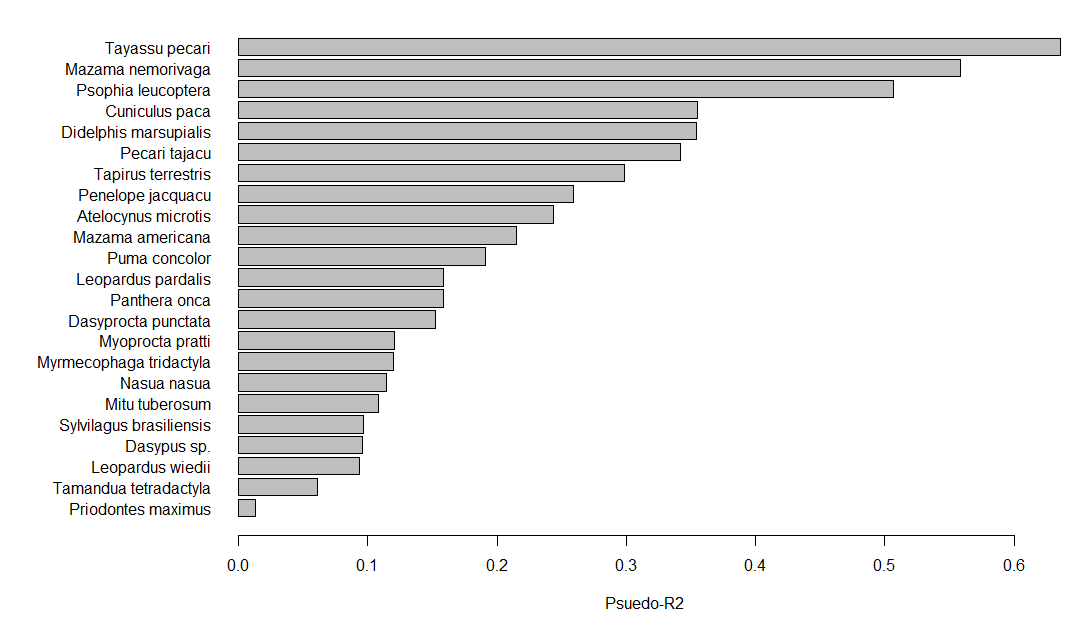
**

*Partitioned R^2^*


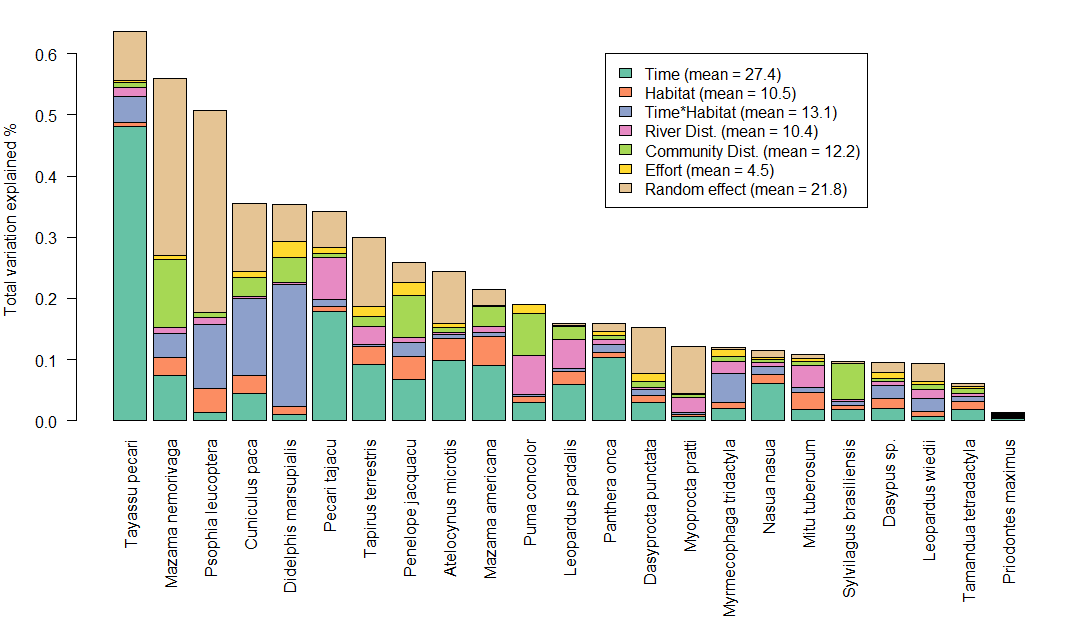


**Appendix E:** Full coefficients for the predictors of species-level counts

**
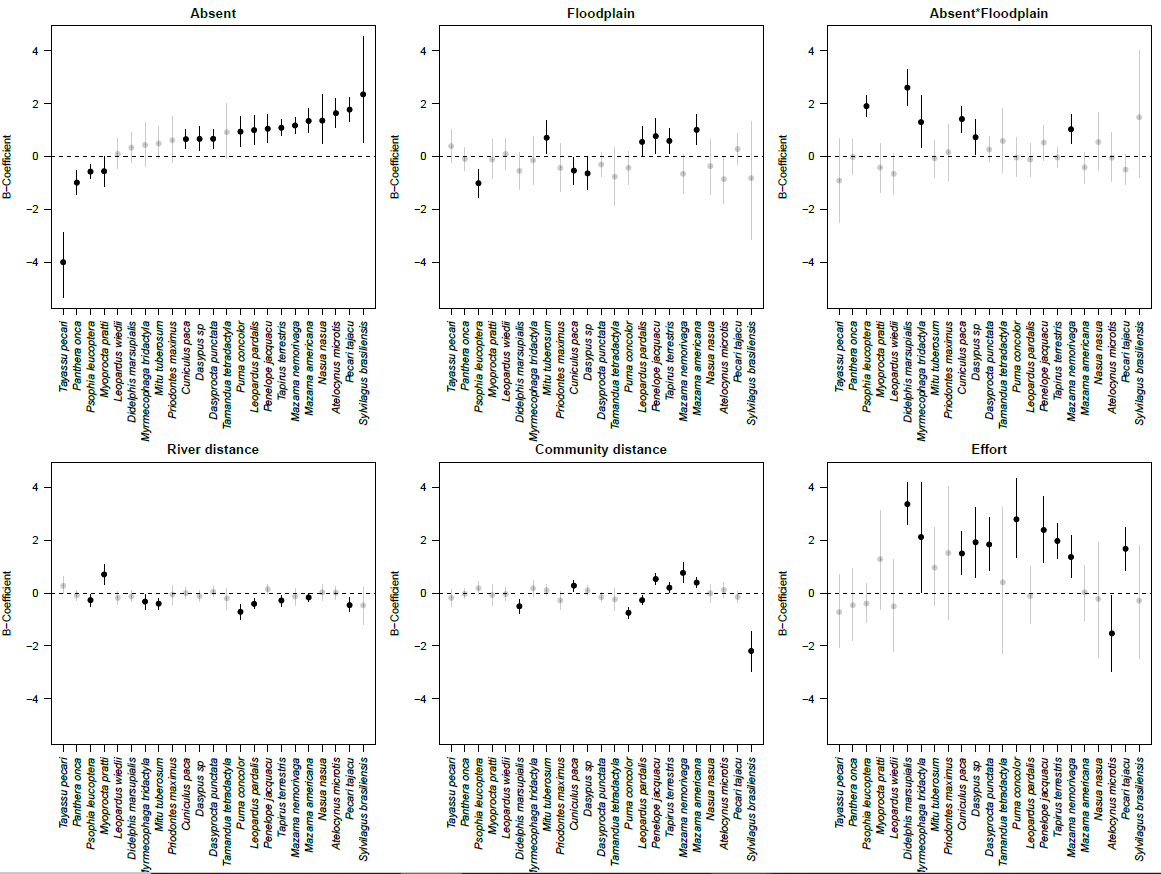
**

**Appendix F:** The relationship between community traits and predictor variables


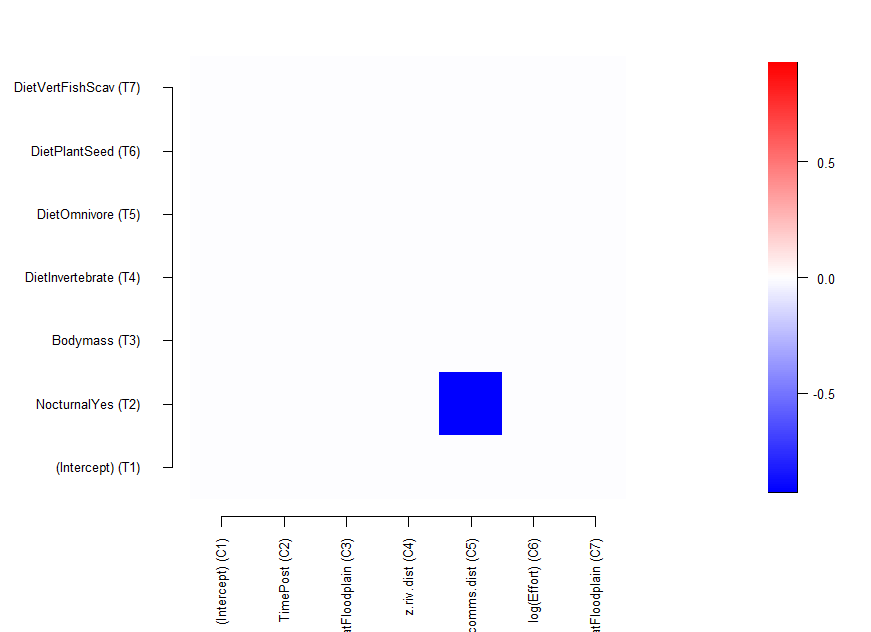


**Appendix G:** Anecdotal evidence of WLP decline in nearby locations

A near-parallel sudden population crash as that observed in LACC was directly observed by co-author JT in his field work spanning decades at Cocha Cashu (~160km to the NW of LACC). This field station lies directly within the well-protected and expansive Manu National Park, and while hunting by indigenous groups within the park is likely to deplete WLP populations locally around villages, these villages are many hours upriver from the field station, suggesting that a hunting-driven collapse in WLP populations is implausible (Ohl-Schacherer et al. 2007). Key visual anecdotal evidence is also noted by JT in his 2012 field season when bloated WLP corpses, covered in vultures, were observed floating down the Manu River, and the ‘stench of death’ emanated from parts of the forest along the river. Co-author AF also observed floating WLP corpses in the Río Los Amigos and the Río Piñi Piñi draining central Manu NP in 2012.

Ohl-Schacherer, J., G. H. Shepard, H. Kaplan, C. A. Peres, T. Levi, and D. W. Yu. 2007. The sustainability of subsistence hunting by Matsigenka native communities in Manu National Park, Peru. Conservation Biology 21:1174–1185.
